# Intrabiliary rupture of liver hydatid cyst: a case report and review of the literature

**DOI:** 10.1186/1757-1626-2-6455

**Published:** 2009-03-10

**Authors:** Serhat Avcu, Özkan Ünal, Halil Arslan

**Affiliations:** 1 Yüzüncü Yil University Medical Faculty, Department of Radiology, 65200 Van, Turkey

## Abstract

Herein, we report a 66 year old woman who was diagnosed to have intrabiliary rupture of liver hydatid cyst with demonstrative computed tomography, magnetic resonance imaging, and magnetic resonance cholangiopancreatography findings, with a review of the literature.

## Introduction

The hydatid cyst is a parasitic disease mainly caused by Echinococcus granulosus. Hydatid cysts are common in the Mediterranean countries, the Middle and Far East, Europe, Asia, South America and Australia. In humans, the most commonly infested organs are the liver and lung [1].

Anaphylactic shock, cyst infection of the biliary tree, and rupture into the peritoneum are the most common complications. Intrabiliary rupture is reported to be seen in a range of 6,1-17% [2]-[4]. In a study which was done when the ultrasonography (US) and computed tomography (CT) were not awailable, this range was reported to be 41% [4]. There are 2 types of intrabiliary rupture: frank and occult (silent) [5].

Abdominal ultrasound, CT and magnetic resonance imaging (MRI) are used to diagnose the complications. Endoscopic retrograde cholangiopancreatography (ERCP) is used extensively nowadays both in the diagnosis and treatment of biliary complications of hepatic hydatid cysts [1]. We report a case of a ruptured hepatic hydatid cyst focusing on the CT, MRI and magnetic resonance cholangiopancreatography (MRCP) diagnosis.

## Case presentation

Sixty-six years old Turkish woman living in eastern Turkey presented to our hospital with complaints of a continuous epigastric and right upper quadrant pain which had a duration of one month. The patient also had nausea, vomitting, loss of appetite, and jaundice. The patient had no previous disease, she is non-alcoholic, and her family history was not significant. On physical examination, diffuse tenderness was noticed at the epigastric and right upper quadrant regions. Laboratory findings revealed increase in serum aspartate transaminase (420 U/L) and alanine aminotransferase levels (180 U/L) (normal up to 50 U/L), elevated bilirubin (5.17 mg/dl), amilase (1292.55 U/L), and gamma glutamyl transferase (154.3 U/L). All the other laboratory examinations were normal.

**Figure 1:**
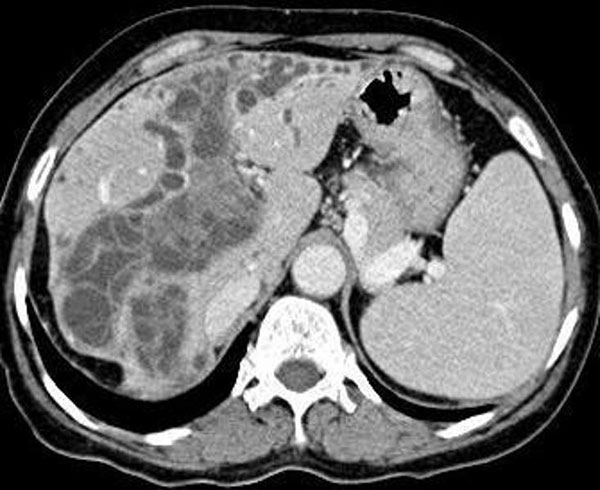
**CT image showing dilated bile ducts which contain lineer hyperdens material corresponding to germinative membranes and other cyst contents**.

**Figure 2:**
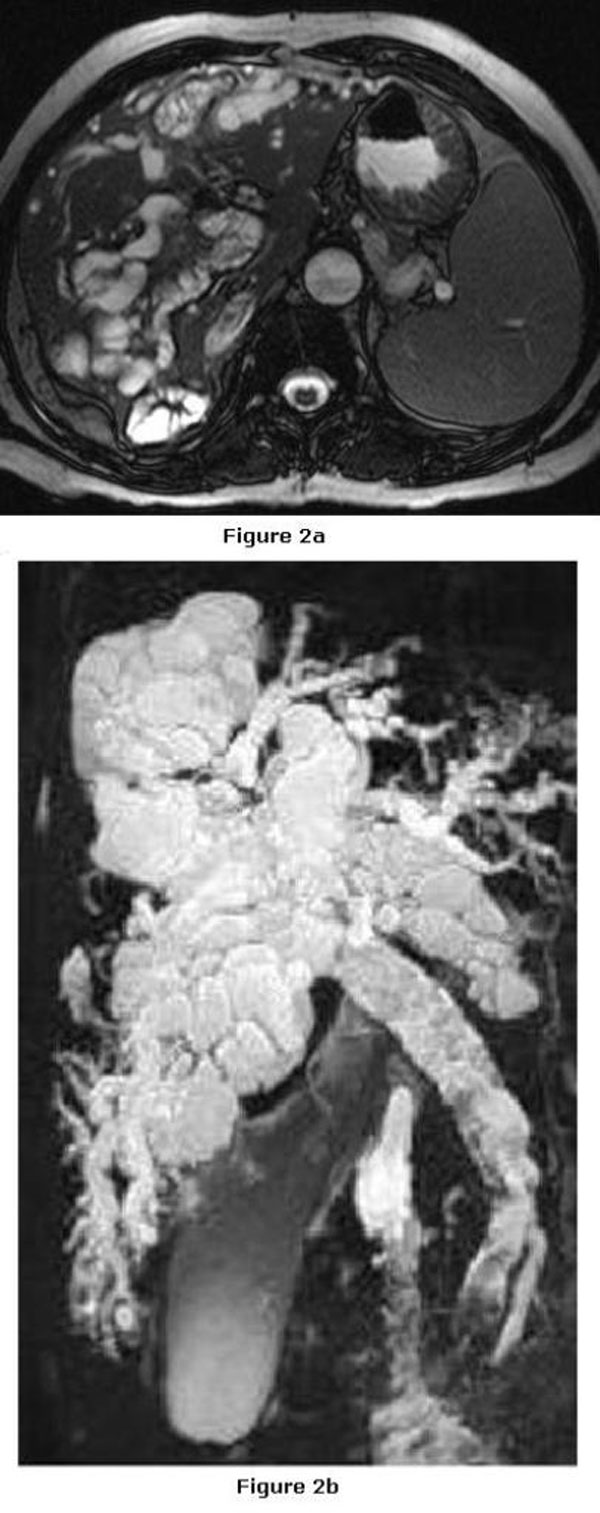
**Axial T2-weighted MRI (a) and MRCP (b) demonstrating extensively dilated hepatic bile ducts and common bile duct filled with hydatic cyst material seen as hypointense lesions**.

On sonography, multiloculated hepatic cystic lesions maximum of 6 cm in diameter and dilatation of the bile ducts were detected. The gallbladder was enlarged, containing stones and echogenic material. The common bile duct was dilated and contained echogenic material inside without an acoustic shadow. The CT scan revealed dilated bile ducts which contained linear hyperdense material corresponding to germinative membranes together with other cyst contents (Figure 1). The MRI and MRCP revealed extensively dilated hepatic bile ducts and common bile duct filled with hydatic cyst material seen as hypointense lesions (Figure 2). The case was interpreted as a ruptured hydatid cyst with biliary obstruction, acute cholangitis and choledochocholecystolithiasis.

Laparotomy, cystectomy, cyst drainage, cholecystectomy, common bile duct exploration, and T-tube drainage were performed surgically. At the operation it was found that the hydatid cyst was ruptured into the bile ducts and there were daughter vesicles in the common bile duct. The gallbladder was hydropic containing multiple stones and cyst material inside.

**Figure 3:**
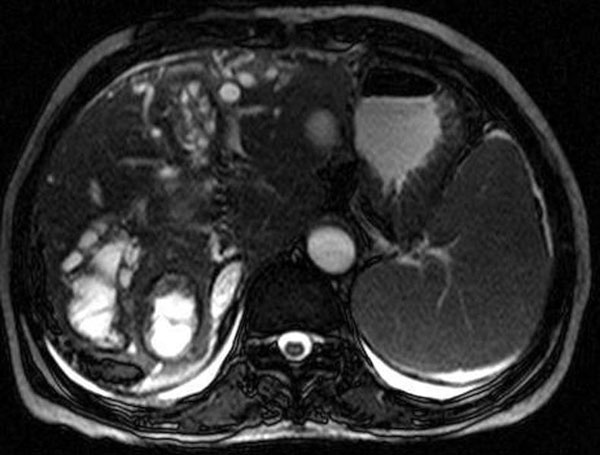
**Axial T2-weighted MRI showing hepatic hydatid cysts and changes secondary to operation**.

All the laboratory findings returned to normal after the operation. Control MRI examinations were performed 6 months after the operation which showed hepatic hydatid cysts and changes secondary to operation (Figure 3).

## Discussion

An increase in pressure inside the cyst precipitated by trauma, effort or violent cough will cause rupture. The rupture may be classified as: *contained rupture* when the endocyst is torn, but the cyst content is confined within the pericyst; *communicating rupture* consists of tear of the endocyst with loss of the cyst content via small biliary ducts and *direct rupture* when a tear of both endocyst and pericyst occurs, allowing the cyst content to spill into the peritoneal or pleural spaces [1].

Compression and displacement of the biliary ducts are frequent. At the point of contact with a biliary duct, a rupture may occur. In case of intrabiliary rupture of liver hydatid cysts, the most apparent signs and symptoms are fever, jaundice, and right upper quadrant pain [6]. The communicating intrabiliary rupture may be: *occult* (seen in 10-37% of patients) or *frank* (seen in 3-17% of patients). The occult rupture is usually silent and may be accompanied by suppuration or can evolve towards a frank rupture. Clinical findings are nonspecific at this stage [5]. In the frank rupture daughter vesicles and fragmented membranes escape into the biliary tree causing obstructive jaundice, acute cholangitis or septicemia. Moreover, acute pancreatitis and acute cholecystitis caused by hydatid material are described in the literature [1,8]-[10]. Diagnosis is easy at this stage.

Although communication between biliary system and cyst occurs in 80%-90% of patients with hepatid hydatic cyst, the incidence of clinical CBC is only 13%-37%. The incidence of frank intrabiliary rupture ranges between 5% and 17%. Although its mortality and morbidity are high, there are no significant problems with the management of CBC. However, occult CBC, which constitutes 10%-37% of cases, is difficult to diagnose, because the symptoms and preoperative radiological findings are unremarkable. CBC is the most common complication of hepatic hydatid cyst, occuring in 14%-25% of cases of postoperative biliary leakage [5]. The patients with ruptured liver hydatid cyst may rarely be asymptomatic [8]. Our case was interpreted as frank intrabiliary rupture.

Before the introduction of US or CT, preoperative diagnosis of the complications of hepatic hydatidosis was difficult and based on clinical manifestations and results of laboratory studies. US and CT may suggest the diagnosis of a frank intrabiliary rupture in most of the cases, whereas MRI provides additional multiplanar images. MRCP provides a characteristic intense rim, daughter cysts, detachment of the membranes, and dilated biliary tree containing hydatid material [11,12].

Potential sites of rupture are peritoneum, bile ducts, pleural space, thorax, and visceral organs such as stomach or duodenum [1].

Surgical management of hepatic hydatid disease has ranged from radical procedures like hepatic resection and total cystopericystectomy to conservative ones like cyst evacuation followed by capsulorrhaphy or external drainage. The aim is to remove the entire disease while minimizing complications. Conservative procedures are safe and technically simple, and are useful in the management of uncomplicated hydatid cysts. However, their main disadvantage is the high frequency of postoperative complications, the most common being bile leak from a cyst-biliary communication and its sequelae like bilio-cutaneous fistulae, bilomas and bile peritonitis (4%-28%) [13].

External biliary fistulae following surgery for liver hydatid disease tend to close spontaneously. In a review of 304 cases, all the 10 external biliary fistulae closed spontaneously over a period of 2-4 months [14]. In another series, 7 of 12 fistulae closed spontaneously, with the maximum time to closure being 38 days. Though most fistulae close spontaneously, the prolonged biliary drainage causes significant morbidity [13].

Most series on hepatic hydatid disease report on a small number of patients with postoperative external biliary fistulae; it is generally accepted that endoscopic management in the form of endoscopic sphincterotomy, with or without stenting or naso-biliary drainage, plays a key role in the management of such patients. Endoscopic sphincterotomy is believed to reduce the high intra-biliary pressure, and promote early closure of these fistulae even in the absence of distal biliary obstruction [15].

As a conclusion, sonographic findings of intrabiliary rupture of hepatic hydatid cysts have been discussed in the literature before, but as far as we know, CT, MRI and MRCP findings are not well demonstrated until now, which made us report our case.

## Abbreviations

TOD: Transomental Defect; CT: Computerised tomography; MRI: Magnetic resonance imaging; MRCP: Magnetic resonance cholangiopancreatography; US: Ultrasonography; ERCP: Endoscopic retrograde cholangiopancreatography; CBC: Complete blood count.

## Consent

Written informed consent was obtained from the patient for publication of this case report and accompanying images. A copy of the written consent is available for review by the Editor-in-Chief of this journal.

## Competing interests

The authors declare that they have no competing interests.

## Authors' contributions

SA, ÖÜ, and HA analyzed and interpreted the patient data regarding the clinical and radiological findings of the patient. All authors were a major contributor in writing the manuscript. All authors read and approved the final manuscript.
